# ‘Scientific Strabismus’ or two related pandemics: coronavirus disease and vitamin D deficiency

**DOI:** 10.1017/S0007114520001749

**Published:** 2020-10-14

**Authors:** Murat Kara, Timur Ekiz, Vincenzo Ricci, Özgür Kara, Ke-Vin Chang, Levent Özçakar

**Affiliations:** 1 Department of Physical Medicine and Rehabilitation, Hacettepe University Medical School, Ankara, Turkey; 2 Department of Physical Medicine and Rehabilitation, Türkmenbaşı Medical Center, Adana, Turkey; 3 Department of Biomedical and Neuromotor Science, Physical and Rehabilitation Medicine Unit, IRCCS Rizzoli Orthopaedic Institute, Bologna, Italy; 4 Geriatrics Unit, Yenimahalle Training and Research Hospital, Yıldırım Beyazıt University, Ankara, Turkey; 5 Department of Physical Medicine and Rehabilitation, National Taiwan University Hospital, Bei-Hu Branch, Taipei, Taiwan

**Keywords:** Coronavirus, Death, Insufficiency, Europe, Acute respiratory syndrome

## Abstract

The WHO has announced the novel coronavirus disease (COVID-19) outbreak to be a global pandemic. The distribution of community outbreaks shows seasonal patterns along certain latitude, temperature and humidity, that is, similar to the behaviour of seasonal viral respiratory tract infections. COVID-19 displays significant spread in northern mid-latitude countries with an average temperature of 5–11°C and low humidity. Vitamin D deficiency has also been described as pandemic, especially in Europe. Regardless of age, ethnicity and latitude, recent data showed that 40 % of Europeans are vitamin D deficient (25-hydroxyvitamin D (25(OH)D) levels <50 nmol/l), and 13 % are severely deficient (25(OH)D < 30 nmol/l). A quadratic relationship was found between the prevalences of vitamin D deficiency in most commonly affected countries by COVID-19 and the latitudes. Vitamin D deficiency is more common in the subtropical and mid-latitude countries than the tropical and high-latitude countries. The most commonly affected countries with severe vitamin D deficiency are from the subtropical (Saudi Arabia 46 %; Qatar 46 %; Iran 33·4 %; Chile 26·4 %) and mid-latitude (France 27·3 %; Portugal 21·2 %; Austria 19·3 %) regions. Severe vitamin D deficiency was found to be nearly 0 % in some high-latitude countries (e.g. Norway, Finland, Sweden, Denmark and Netherlands). Accordingly, we would like to call attention to the possible association between severe vitamin D deficiency and mortality pertaining to COVID-19. Given its rare side effects and relatively wide safety, prophylactic vitamin D supplementation and/or food fortification might reasonably serve as a very convenient adjuvant therapy for these two worldwide public health problems alike.

## Graphical abstract

**Figure:**
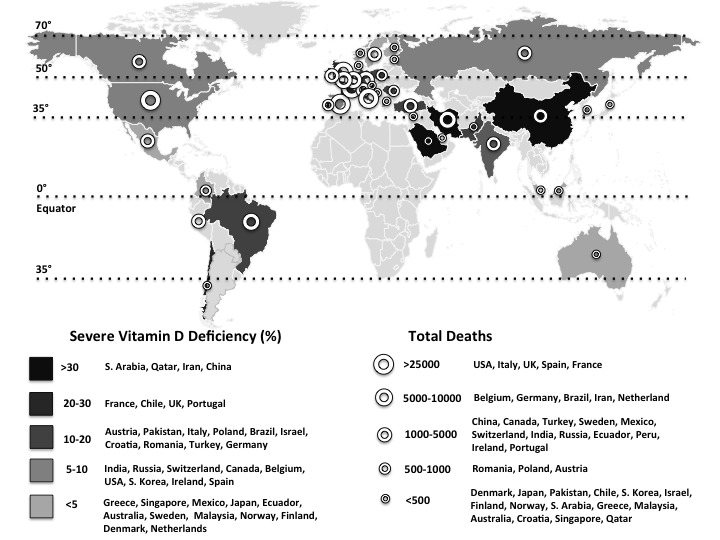

On 11 March 2020, the WHO announced the novel coronavirus disease (COVID-19) outbreak to be a global pandemic^(1)^. The spread of COVID-19 is becoming unstoppable, and as of 15 May, more than 4 500 000 people have been infected and more than 300 000 people have died (Fig. 1)^(1)^. The severe acute respiratory syndrome coronavirus 2 (SARS-Cov-2) is the pathogen of COVID-19. SARS-CoV-2, classified into two β coronaviruses, is an enveloped, positive-sense and single-stranded RNA virus of about 30 kb. The life cycle of the virus with the host comprises mainly five steps as follows: attachment, penetration, biosynthesis, maturation and release. Once SARS-CoV-2 attaches to the host receptors, it penetrates the cells via endocytosis/membrane fusion. Herein, angiotensin-converting enzyme 2 is the entry and functional receptor of SARS-CoV-2. It has been shown that the spike for SARS-CoV-2, structural membrane proteins formed by the *trans*-membrane trimetric glycoprotein protruding from the viral surface, also binds to angiotensin-converting enzyme 2. After the viral contents are released inside the host cells, viral RNA enters the nucleus to replicate. As for the biosynthesis, viral mRNA is used to make viral proteins. The new viral particles are formed in the maturation step and then released^(2)^.

**Fig. 1.**
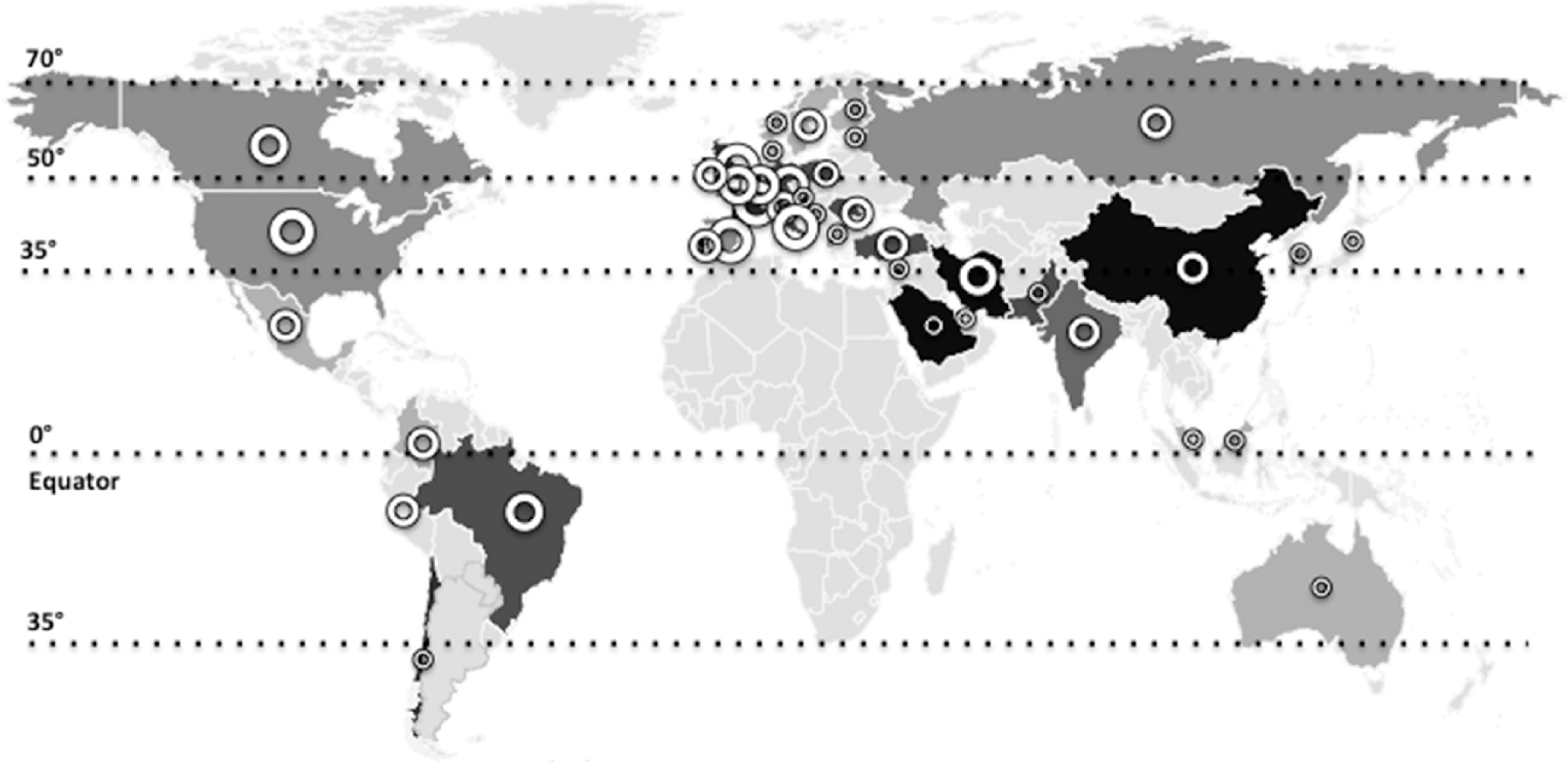
The world map illustrates the total deaths and percentage of severe vitamin D deficiency in countries most commonly affected by COVID-19^(1,5–43)^. Severe vitamin D deficiency (%): (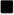), >30 (South Arabia, Qatar, Iran, China); (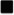), 20–30 (France, Chile, UK, Portugal); (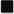), 10–20 (Austria, Pakistan, Italy, Poland, Brazil, Israel, Croatia, Romania, Turkey, Germany); (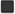), 5–10 (India, Russia, Switzerland, Canada, Belgium, USA, South Korea, Ireland, Spain); (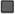), <5 (Greece, Singapore, Mexico, Japan, Ecuador, Australia, Sweden, Malaysia, Norway, Finland, Denmark, Netherlands). Total deaths: (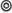), >25 000 (USA, UK, Italy, France, Spain); (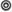), 5000–10 000 (Brazil, Belgium, Germany, Iran, The Netherlands, Canada); (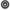), 1000–5000 (China, Mexico, Turkey, Sweden, India, Ecuador, Russia, Peru, Switzerland, Ireland, Portugal, Romania); (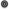), 500–1000 (Poland, Pakistan, Japan, Austria, Denmark); (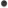), <500 (Chile, Finland, Saudi Arabia, Israel, South Korea, Norway, Greece, Malaysia, Australia, Croatia, Singapore, Qatar).

Angiotensin-converting enzyme 2 plays an important role for the interaction between the classical and non-classical pathway of the renin angiotensin system. The former acts through the angiotensin II type 1 receptors, and its increased activity leads to fibrosis, inflammation and angiogenesis. The latter acts through the Mas receptors and has opposing effects to the angiotensin II type 1 receptors^(3)^. Angiotensin-converting enzyme 2 is expressed by the epithelial cells of lungs, intestines, kidneys and blood vessels; therefore, the aforementioned tissues/organs are vulnerable to SARS-CoV-2 infection^(4)^. Additionally, activation of the renin angiotensin system is significantly associated with increased morbidity and mortality as in hypertension^(3)^.

On the other hand, vitamin D deficiency has also been described as pandemic and a global public health problem, especially in Europe (Table 1)^(5–43)^. Regardless of age, ethnicity and latitude, recent data showed that 40 % of Europeans are vitamin D deficient (25-hydroxyvitamin D (25(OH)D) levels <50 nmol/l), and 13 % are severely deficient (25(OH)D < 30 nmol/l)^(44)^. According to regression analyses, a quadratic relationship was found between the prevalences of vitamin D deficiency in most commonly affected countries by COVID-19 and the latitudes (Fig. 2). Interestingly, vitamin D deficiency is more common in the subtropical and mid-latitude countries than the tropical and high-latitude countries. Contrary to the expectation, the most commonly affected countries with severe vitamin D deficiency are from the subtropical (Saudi Arabia 46 %; Qatar 46 %; Iran 33·4 %; Chile 26·4 %) and mid-latitude (France 27·3 %; Portugal 21·2 %; Austria 19·3 %) regions. On the other hand, severe vitamin D deficiency was found to be nearly 0 % in some high-latitude countries (e.g. Norway, Finland, Sweden, Denmark and Netherlands). The low prevalences of severe vitamin D deficiencies in high-latitude countries (except for the UK; 23·7 %) can possibly be attributed to the high awareness of vitamin D deficiency, high amount of vitamin D supplementation, food fortification and health policies as well^(44)^. Indeed, as the main source of vitamin D is exposure of the skin to sun (UV-B), it has long been supposed that living in a sunny country guarantees sufficient vitamin D levels. However, there is increasing evidence that vitamin D deficiency may have been underestimated/ignored in low latitude, even in tropical countries^(45)^.

**Table 1.** Available data for vitamin D deficiency among older adults in countries most commonly affected by COVID-19

Rank*	Region/country	Latitude	*n*	Age (years): mean (±sd) or range	25(OH)D deficiency (%)		Reference and year
					<25 nmol/l†	<50 nmol/l	
	Region of the Americas						
1	USA	Mid latitude (38°)	4363	76±5	7 (≤30)	32	Eymundsdottir *et al.* ^(5)^ 2020
6	Brazil	Subtropics (23°)	908	73±5	14·4	58·0	Lopes *et al.* ^(6)^ 2014
13	Peru	Tropics (12°)	204	39±11	N/A	29·4	Pastor *et al.* ^(8)^ 2019
14	Canada	Mid latitude (45°)	11 336	3–79	7·4 (<30)	36·8	Sarafin *et al.* ^(7)^ 2015
18	Mexico	Tropics (19°)	585	41±15	2·0	43·6	Clark *et al.* ^(10)^ 2015
20	Chile	Subtropics (33°)	686	≥65	26·4 (<30)	64·9	Solis-Urra *et al.* ^(11)^ 2019
21	Ecuador	Tropics (0°)	2374	71±8	N/A	21·6	Orces *et al.* ^(9)^ 2015
	European region						
2	Spain	Mid latitude (41°)	312	≥85	14·4	N/A	Formiga *et al.* ^(12)^ 2014
3	Russia	High latitude (59°)	1664	3–75	8·0 (<30)	45·7	Karonova *et al.* ^(17)^ 2016
4	UK	High latitude (54°)	6004	<50	23·7 (<30)	55·3	Aspell *et al.* ^(14)^ 2019
5	Italy	Mid latitude (41°)	2640	65–98	10·6	21·6	Veronese *et al.* ^(13)^ 2014
7	France	Mid latitude (44°)	697	73±4	27·3	55·9	Cougnard-Grégoire *et al.* ^(15)^ 2015
8	Germany	High latitude (51°)	1671	60–85	10·7	48·6	Vetter *et al.* ^(16)^ 2020
9	Turkey	Mid latitude (37°)	1161	18–90	12·9	75·5	Öztürk *et al.* ^(18)^ 2017
15	Belgium	High latitude (50°)	697	32–53	7·3	51·1	Hoge *et al.* ^(19)^ 2015
17	Netherlands	High latitude (52°)	450	65–93	0·0	2·0	Ten Haff *et al.* ^(20)^ 2019
22	Switzerland	Mid latitude (47°)	1291	≥60	8·0	39·2	Sakem *et al.* ^(21)^ 2013
23	Sweden	High latitude (56°)	995 (W)	80–81	0·0	16·0	Buchebner *et al.* ^(23)^ 2014
24	Portugal	Mid latitude (38°)	3092	≥18	21·2	66·6	Duarte *et al.* ^(22)^ 2020
27	Ireland	High latitude (53°)	1118	18–84	6·0	45·0	Cashman *et al.* ^(24)^ 2013
28	Poland	High latitude (51°)	5775	16–90	16·0	65·8	Płudowski *et al.* ^(26)^ 2016
30	Romania	Mid latitude (46°)	14 052	37–62	13·2	52·0	Niculescu *et al.* ^(27)^ 2017
32	Austria	Mid latitude (47°)	161	65–80	19·3	64·0	Elmadfa *et al.* ^(25)^ 2017
34	Denmark	High latitude (56°)	3409	19–72	0·0	23·6	Cashman *et al.* ^(28)^ 2015
35	Norway	High latitude (69°)	12 817	30–87	0·3	18·6	Cashman *et al.* ^(29)^ 2016
38	Finland	High latitude (64°)	4102	29–77	0·2	6·6	Cashman *et al.* ^(28)^ 2015
39	Greece	Mid latitude (37°)	181 (M)	20–50	4·4	50·3	Kassi *et al.* ^(30)^ 2015
40	Croatia	Mid latitude (45°)	120 (W)	61±9	14·2 (<30)	63·3	Laktasic *et al.* ^(31)^ 2010
	Western Pacific Region						
11	China	Subtropics (31°)	2180	>65	30·6 (<30)	70·3	Wei *et al.* ^(32)^ 2019
26	Singapore	Tropics (1°)	504	45–74	N/A	14·0	Robien *et al.* ^(33)^ 2013
31	Japan	Mid latitude (38°)	9084	40–74	N/A	53·6	Nakamura *et al.* ^(34)^ 2015
33	South Korea	Mid latitude (36°)	4107 (W)	50–79	6·6 (<37·5)	67·4	Shin *et al.* ^(35)^ 2015
36	Australia	Subtropics (34°)	2413	51±17	0·9	22·7	Gill *et al.* ^(36)^ 2014
37	Malaysia	Tropics (3°)	63 (M)	≥60	0·0	17·5	Chin *et al.* ^(37)^ 2014
	Eastern Mediterranean region						
10	Iran	Subtropics (30°)	370	≥35	33·4	61·9	Khosravi-Boroujeni *et al.* ^(38)^ 2017
16	Saudi Arabia	Subtropics (24°)	3475	47±16	46·0	76·8	Alfawaz *et al.* ^(39)^ 2014
19	Pakistan	Subtropics (30°)	858	18–60	N/A	58·4	Mehboobali *et al.* ^(40)^ 2015
25	Qatar	Subtropics (25°)	547	49±13	46·0	N/A	El-Menyar *et al.* ^(42)^ 2012
29	Israel	Subtropics (31°)	198 834	60 (median)	14·4	49·9	Saliba *et al.* ^(41)^ 2012
	South-East Asia region						
12	India	Tropics (12°)	149	≥46	8·7 (<30)	48·3	Mechenro *et al.* ^(43)^ 2018

*n*, Number; 25(OH)D, 25-hydroxyvitamin D; N/A, not applicable; W, women; M, men.

* The most commonly infected countries and regions with COVID-19 in descending order.

† Percentages of severe vitamin D deficiency.

**Fig. 2.**
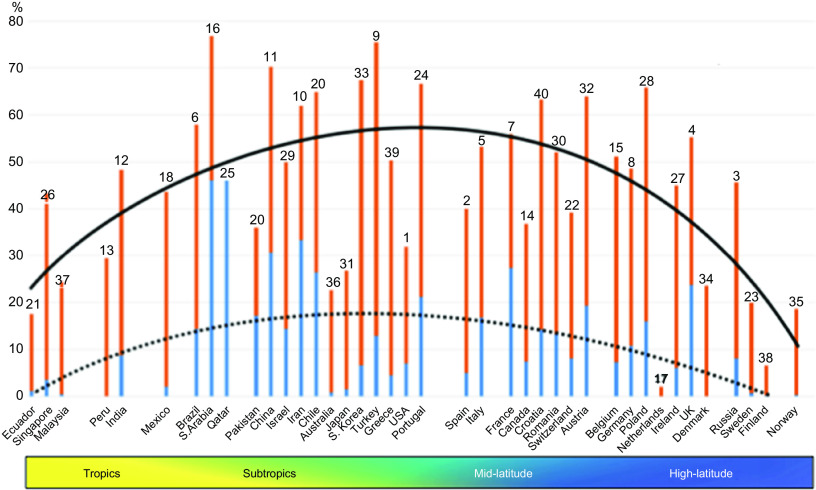
The histogram shows the prevalence of vitamin D deficiency (<50 nmol/l) and severe deficiency (<25 nmol/l) among the forty countries most commonly affected by COVID-19. The number above each column represents the country’s position in the world ranking concerning the number of total cases of infections. The colour band is a graphical representation of the four main climatic areas in the world. Regression lines show the prevalence of overall (solid black line) and severe (dotted line) vitamin D deficiencies. (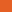), Vitamin D deficiency; (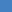), severe vitamin D deficiency.

The risks for vitamin D deficiency encompass obesity, elderly, lack of proper sun (UV-B) exposure, dark skin, smoking, living with air pollution and the presence of co-morbid diseases such as infection, cancer, CVD, chronic respiratory disease, osteoporosis, sarcopenia and diabetes mellitus^(46,47)^. Further, it is known that severe vitamin D deficiency dramatically increases the risk of mortality, infections and many other diseases. As such, it should indisputably be prevented whenever detected/possible^(46)^.

Vitamin D hormone has important functions – including immunomodulant, anti-inflammatory and anti-infective roles^(47)^. It acts via monocyte and cell-mediated immunity stimulation, suppression of lymphocyte proliferation, antibody production and cytokine synthesis^(48)^. Human lung cells are able to intracellularly convert the inactive 25(OH)D to its active form 1,25(OH)D which reduces proinflammatory cytokines and increases peptides (e.g. the innate antimicrobial peptide cathelicidin)^(48)^. Cathelicidin has direct antiviral activity against enveloped respiratory viruses such as hepatitis B, influenza, respiratory syncytial virus and possibly the COVID-19 as well^(48)^. Other than the above-mentioned functions, vitamin D has also anti-fibrotic effects. The renin-inhibiting activity and down-regulation of the renin angiotensin system activity seem to be the beneficial effects of vitamin D. Moreover, vitamin D has been shown to suppress angiotensinogen and regulate its expression^(49)^.

The distribution of community outbreaks shows seasonal patterns along certain latitude, temperature and humidity, that is, similar to the behaviour of seasonal viral respiratory tract infections. It has been reported that COVID-19 displays significant spread in mid-latitude (35–50° N′) regions and/or in those with an average temperature of 5–11°C and low humidity (Fig. 1)^(1,50)^. Coronaviruses are very stable at 4°C (viable for up to 3 d) and can survive at −20°C (for up to 2 years)^(1)^. Depending on some parameters (e.g. temperature, humidity and sunlight), they can live on different surfaces for a few days. They are thermolabile; decreased sunlight, low temperatures and less humidity seem to be favourable for COVID-19^(1)^. Although natural UV (UV-C) from the sunlight may not be strong enough to kill COVID-19, its antimicrobial efficacy has long been shown to inactivate, thus preventing the transmission of airborne-mediated infections such as influenza and tuberculosis^(51)^. Further, UV-B from the sun can induce endogenous synthesis of vitamin D in the skin – being the main source of vitamin D other than the dietary intake or supplementation. These factors might possibly be explanatory as regards the low prevalence of COVID-19 in subtropical and southern countries.

Patients infected with COVID-19 have higher mortality rates if they are older, that is, 8·0 % (70–79 years) and 14·8 % (>80 years). The similar rates for co-morbid conditions are as 10·5 % (CVD), 7·3 % (diabetes mellitus), 6·3 % (chronic respiratory disease), 6·0 % (hypertension) and 5·6 % (cancer)^(52)^. Older adults with any of these co-morbid diseases are at high risk for COVID-19 infection – especially in the presence of severe vitamin D deficiency^(53)^. To this end, since there is positive/strong evidence concerning the effects of vitamin D against viral respiratory infections, it would not be unsound to say that vitamin D supplementation may decrease viral induction and inflammatory genes, and incidence/severity of respiratory tract infections^(48)^. In this sense, a meta-analysis of twenty-five randomised controlled trials showed that vitamin D supplementation has a preventive effect against acute respiratory tract infections and that the benefit is higher in those subjects receiving daily or weekly vitamin D without additional bolus doses, and in those having severe vitamin D deficiency at baseline^(54)^.

Although vitamin D was primarily recognised for bone metabolism, increasing evidence indicates its proper function for nearly every tissue in the body including brain, heart, lung, muscle, immune system and skin^(55)^. Therefore, the treatment of vitamin D deficiency would be vital for several diseases including cardiovascular and neurological disorders, cancers, autoimmune diseases and infections as well^(55)^. Likewise, a recent review recommended that in people at risk of influenza/COVID-19 infection, 250 μg/d of vitamin D_3_ for a few weeks (or a month),that is, to rapidly increase the 25(OH)D concentrations and then 125 μg/d in the follow-up can be considered^(47)^. The target should be to raise its value above 40–60 ng/ml. Additionally, the authors also suggested higher vitamin D_3_ doses for infected patients with COVID-19. For sure, attention should be paid not to take high calcium supplementation for potential risk of hypercalcaemia while taking high doses of vitamin D_3_. Needless to say, as vitamin D is synthesised mainly in the skin, sun (UV-B) exposure (15–20 min daily) inducing the light pink colour of minimal erythema would be the natural way of production and activation of vitamin D by keratinocytes^(55)^.

Accordingly, presenting this paper, we would like to call attention to the possible association between severe vitamin D deficiency and mortality pertaining to COVID-19. Given its rare side effects and relatively wide safety, prophylactic vitamin D supplementation and/or food fortification might reasonably serve as a very convenient and incomparable/invaluable adjuvant therapy for these two worldwide public health problems alike.
